# Nurses' Leagues and Matrons' Letters

**Published:** 1916-10-07

**Authors:** 


### THE MATRONS' AND SISTERS' DEPARTMENT.

### NURSES' LEAGUES AND MATRONS' LETTERS.

One of the most important and onerous of the duties of a matron is to keep in touch with her nurses after they have passed out of their training-school. The foundation of Nurses' Leagues is a valuable way of preventing nurses from feeling that they have no longer any claim on their hospital, but such Leagues do not in the least detract from the obligation which rests on all matrons to instil into their nurses the conviction that they will be sure of a welcome, of advice and help at any time they may need it. A nurse should feel when she leaves her training-school that the matron will never be too busy or too absorbed in other duties to lend an interested ear to the doings of the nurses she has trained, whether those doings be great or small; to sympathise in their sorrows or rejoice in their joys. The feeling the matron should try to cultivate is that of a nurse long since married, with grown-up children of her own, who wrote of her old matron: "To me she will always be the dearest and best matron in the world, and my children all know her as their 'Granny Matron.'"

Nurses should be encouraged to write frequently or regularly to their old matron. It is clearly impossible for matrons to answer all letters immediately they are received, and nurses do not expect it. The best way is for a postcard, not necessarily written by the matron herself, to be sent acknowledging the letter, saying that the matron hopes to reply to it later, and there should always be some friendly informal remark such as "Matron is glad you like your new work," or "is sorry you have not been well," to give the little personal touch which shows the letter has been read with interest. However severe a nurse may have thought her matron during her training, she should always feel that she can turn to the head of her training-school in any trouble and be sure of a champion, and that no other person will ever know what passes between them. The matron need never expect her nurses to write to her for advice if they have reason to think that a letter revealing any difficulty is likely to be given to a secretary to answer or be left lying about in a much-frequented office. Perhaps only those who have experienced the joy of receiving a friendly note or postcard in the familiar handwriting can realise all it means to a lonely nurse who is struggling to maintain the methods taught her under much easier conditions in her old hospital school; but the appreciation is rarely expressed, and the overworked matron is often constrained to leave this part of her work undone, thinking it less useful than other more obvious duties.

Some matrons keep an informal register as well as the official one, in which a page is devoted to each nurse, on which is noted the date of the last letter received from her and the facts contained in it. If any nurse has allowed more than a year to elapse without writing, a postcard is sent asking for news of her. Other matrons write a letter which is lithographed and sent to each graduate nurse on the register, telling all hospital news: new buildings, alterations, and promotions, marriages, etc., of any other old members of the training-school. Nurses are shy people; they are often very diffident about writing to the matron about their doings when there is no apparent reason, and yet do not like to be thought to write only when they want something—an address of another nurse, a testimonial, or advice about new work. The sending of even a lithographed letter keeps the nurse in touch with her hospital, and by the necessity which the recipient should feel of acknowledging its receipt provides her with a reason—it may only be an excuse—for unburdening herself to Matron. And the response to such or any letters is the opportunity of which Matron, however busy she may be, should always take advantage.

